# Multilocus phylogeny reveals taxonomic misidentification of the *Schizoporaparadoxa* (KUC8140) representative genome

**DOI:** 10.3897/mycokeys.38.28497

**Published:** 2018-08-28

**Authors:** Javier Fernández-López, María P. Martín, Margarita Dueñas, M. Teresa Telleria

**Affiliations:** 1 Departmento de Micología, Real Jardín Botánico, RJB-CSIC, Plaza de Murillo 2, 28014 Madrid, Spain Real Jardín Botánico Madrid Spain

**Keywords:** Hymenochaetales, phylogenetic analyses, taxonomy, white-rot fungi, *
Xylodon
*

## Abstract

*Schizoporaparadoxa*, current name *Xylodonparadoxus*, is a white-rot fungus with certain useful biotechnological properties. The representative genome of *Schizoporaparadoxa* strain KUC8140 was published in 2015 as part of the 1000 Fungal Genomes Project. Multilocus phylogenetic analyses, based on three nuclear regions (ITS, LSU and *rpb*2), confirmed a misidentification of *S.paradoxa* strain KUC8140 which should be identified as *Xylodonovisporus*. This wrong identification explains the unexpected geographical distribution of *S.paradoxa*, since this species has a European distribution, whereas the strain KUC8140 was recorded from Korea, Eastern Asia.

## Introduction

The genus *Schizopora* Velen., currently synonymous with *Xylodon* (Pers.) Fr. (Riebesehl and Langer 2017), includes white-rot fungi that play an important role in ecosystem processes as a wood decomposer. The description and identification of *Xylodon* (=*Schizopora*) species, based on morphological characters, has led to inaccuracies due to a lack of clear diagnostic characters and it has been assumed that many *Xylodon* species have a worldwide distribution (Paulus et al. 2000). However, during the last decade, it has been pointed out that fungal cosmopolitanism could be the result of the application of a morphological species recognition criterion and not the result of an actual biogeographical pattern (Taylor et al. 2006). Moreover, phylogenetic analyses have revealed an undescribed species diversity masked by the morphological species recognition approach (Taylor et al. 2000).

The representative genome of *Schizoporaparadoxa* strain KUC8140, current name *Xylodonparadoxus* (Schrad.) Chevall., was sequenced in 2015 as part of the 1000 Fungal Genomes Project (http://jgi.doe.gov/fungi) (Min et al. 2015); this strain was collected from an oak forest in Korea. Usually *X.paradoxus* has been associated with late stages of wood decay, mainly in deciduous forests and shows useful biotechnological properties for bioremediation, such as tolerance to heavy metals or dye decolourising activity (Lee et al. 2014). It has been recorded around the world; however, available genetic data point to a European distribution (Paulus et al. 2000). Within the framework of a broader study of *Xylodon* through molecular approaches, the taxonomic identity of the strain KUC8140 has been assessed.

## Materials and methods

In order to infer the taxonomic position of the strain KUC8140, phylogenetic relationships of six *Xylodon* species were addressed. DNA from specimens of *X.paradoxus*, *X.quercinus* (Pers.) Gray, *X.nothofagi* (G. Cunn.) Hjorstam & Ryvarden, *X.raduloides* Riebesehl & E. Langer, *X.flaviporus* (Ber. & M.A. Curtis ex Cooke) Riebesehl & E. Langer and *X.ovisporus* (Corner) Riebesehl & E. Langer was extracted from herbaria specimens and culture collections (Table 1). Three specimens of the sister genus *Lyomyces* P. Karst. were included as outgroup in the phylogenetic analyses (Table 1). DNA isolation was performed using DNeasy™ Plant Mini Kit (Qiagen, Valencia, California, USA) following the manufacturer’s instructions. Three nuclear regions were amplified and sequenced: nuclear ribosomal internal transcribed spacer (ITS, fungal barcoding; Schoch et al. 2012), nuclear large ribosomal subunit (LSU) and the second largest subunit of RNA polymerase II (*rpb*2). Direct Polymerase chain reactions (PCRs) were performed to obtain sequences from ITS and LSU with the pair of primers ITS5/ITS4 (White et al. 1990) and LR0R/LR5 (Rehner and Samuels 1994), respectively. Nested-PCRs were done to obtain amplifications of *rpb*2 fragments, using RPB2-5F/RPB2-7.1R (Liu et al. 1999, Matheny 2005) for the first amplification followed by RPB2-6F/RPB2-7R2 (Matheny et al. 2007), using 1 μl of the first PCR as target DNA. Amplifications were undertaken using illustra™ PuReTaq™ Ready-To-Go™ PCR beads (GE Healthcare, Buckinghamshire, UK) as described in Winka et al. (1998), following thermal cycling conditions in Martín and Winka (2000). Negative controls lacking fungal DNA were run for each experiment to check for contamination. Amplifications were assayed by gel electrophoresis in 2% Pronadisa D-1 Agarose (Lab. Conda, Torrejón de Ardoz, Spain). Amplified DNA fragments were purified from the agarose gel using the Wizard SV Gel and PCR Clean-Up System (Promega Corporation, Madison, WI, USA) and sent to Macrogen Korea (Seoul, Korea) for sequencing. Primers, used for sequencing, were those used for PCR amplifications. Additional searches for the six *Xylodon* species in EMBL/GenBank/DDBJ databases were performed in order to complete the molecular information available for this genus.

**Table 1.** Specimen information, GenBank accession numbers and genome BLAST searches (ID) used in this study. New sequences generated in this study are indicated in bold. n.d.: no data.

Species	Specimen voucher	Country	GenBank accession number		
			ITS	LSU	rpb2
* Lyomyces crustosus *	HHB 10401	USA	**MH260068**	**MH260061**	**MH259316**
	HHB 13100	USA	**MH260069**	**MH260062**	**MH259317**
	UC 2022841	USA	KP814310	n.d.	n.d.
* Xylodon flaviporus *	ICMP 13836	Taiwan	AF145585	n.d.	n.d.
	MA-Fungi 79440, 12094IS	Germany	**MH260071**	**MH260066**	**MH259319**
* Xylodon nothofagi *	ICMP 13839	New Zealand	AF145582	**MH260064**	**MH259322**
	PDD 91630, BCP 3306	New Zealand	GQ411524	n.d.	n.d.
* Xylodon ovisporus *	ICMP 13835	Taiwan	AF145586	**MH260063**	**MH259320**
	ICMP 13837	Taiwan	AF145587	n.d.	n.d.
* Xylodon paradoxus *	FCUG 2425	Russia	AF145577	n.d.	n.d.
	MA-Fungi 70444, 11060MD	France	**MH260070**	**MH260065**	n.d.
	MA-Fungi 81294, 13833MD	France	**MH260072**	n.d.	**MH259318**
* Xylodon quercinus *	H 6013352	Finland	KT361632	n.d.	n.d.
	MA-Fungi 91311, 1JFL	Spain	**MH260073**	**MH260067**	**MH259321**
* Xylodon raduloides *	ICMP 13833	Australia	AF145580	**KY962853**	n.d.
	MA-Fungi 75310, GP2291	Spain	**KY962825**	**KY962864**	**KY967055**
* Schizopora paradoxa *	KUC8140	Korea	**ID14957398**	**ID14957349**	**ID1495735**

Using the BLAST tool from the JGI portal, ITS, LSU and *rpb*2 sequences were extracted from the KUC8140 strain genome (https://genome.jgi.doe.gov/pages/blast-query.jsf?db=Schpa1). The same regions from *X.paradoxus* specimens FCUG-2425, MA-Fungi 70444 and MA-Fungi 81294 were used as reference sequences for BLAST searches, respectively (Table 1). For ITS and LSU, custom search settings were used (blastn; all databases; Expect = 1*10^-3^; Word size = 11; Filter low complexity regions; Scoring matrix = PAM30; ITS Job ID = 14957398; LSU Job ID = 14957349). For *rpb*2, default BLAST settings were used (blastn; assembly database; Expect = 1*10^-5^; Word size = 11; Filter low complexity regions; Scoring matrix = BLOSUM62; *rpb*2 Job ID = 14957357). The best scoring sequence from the *S.paradoxa* KUC8140 strain genome for each region was extracted and downloaded.

Raw sequence data were processed and assembled with Geneious version 9.0.2. (Kearse et al. 2012). Two individual datasets, ITS-LSU concatenated and *rpb*2, were created to compare the KUC8140 strain with other *Xylodon* species. The combination of novel, GenBank and KUC8140 sequences for each dataset were aligned in Geneious 9.0.2 with the MAFFT nucleotide sequence alignment function (Katoh and Standley 2013). The automatic alignments were reviewed manually through Geneious 9.0.2.

Phylogenetic tree estimation for each alignment was performed using Maximum Likelihood (ML) and Bayesian Inference (BI). ML and bootstrapping analyses were conducted in RAxML (Stamatakis 2006), using default parameters established in the CIPRES web portal (http://www.phylo.org/portal2/; Miller et al. 2010) and calculating bootstrap statistics from 1000 replicates. Bayesian inference analyses were implemented in BEAST v2.4.3 (Drummond and Rambaut 2007). Site model partition was selected using jModelTest2 (Darriba et al. 2012) and defined using BEAUti v2.4.3 interface. HKY and GTR substitution models were selected for ITS+LSU and *rpb*2 alignments, respectively, as the closest available in BEAST from the results obtained in jModelTest2. We used relative timing with an uncorrelated lognormal relaxed clock by calibrating the tree with a value of 1 in the root for the *Xylodon* clade. Birth Death model was used as a tree prior. One MCMC run was specified for 50 million generations, sampling every 5000th generation. Results were visualised in Tracer v.1.6 (Rambaut et al. 2018) to evaluate whether the effective sample size (ESS) values were above 200. The trees obtained were summarised in a maximum clade credibility tree by TreeAnnotator v.1.7. with a burn-in of 5000.

## Results and discussion

**Figure 1.**
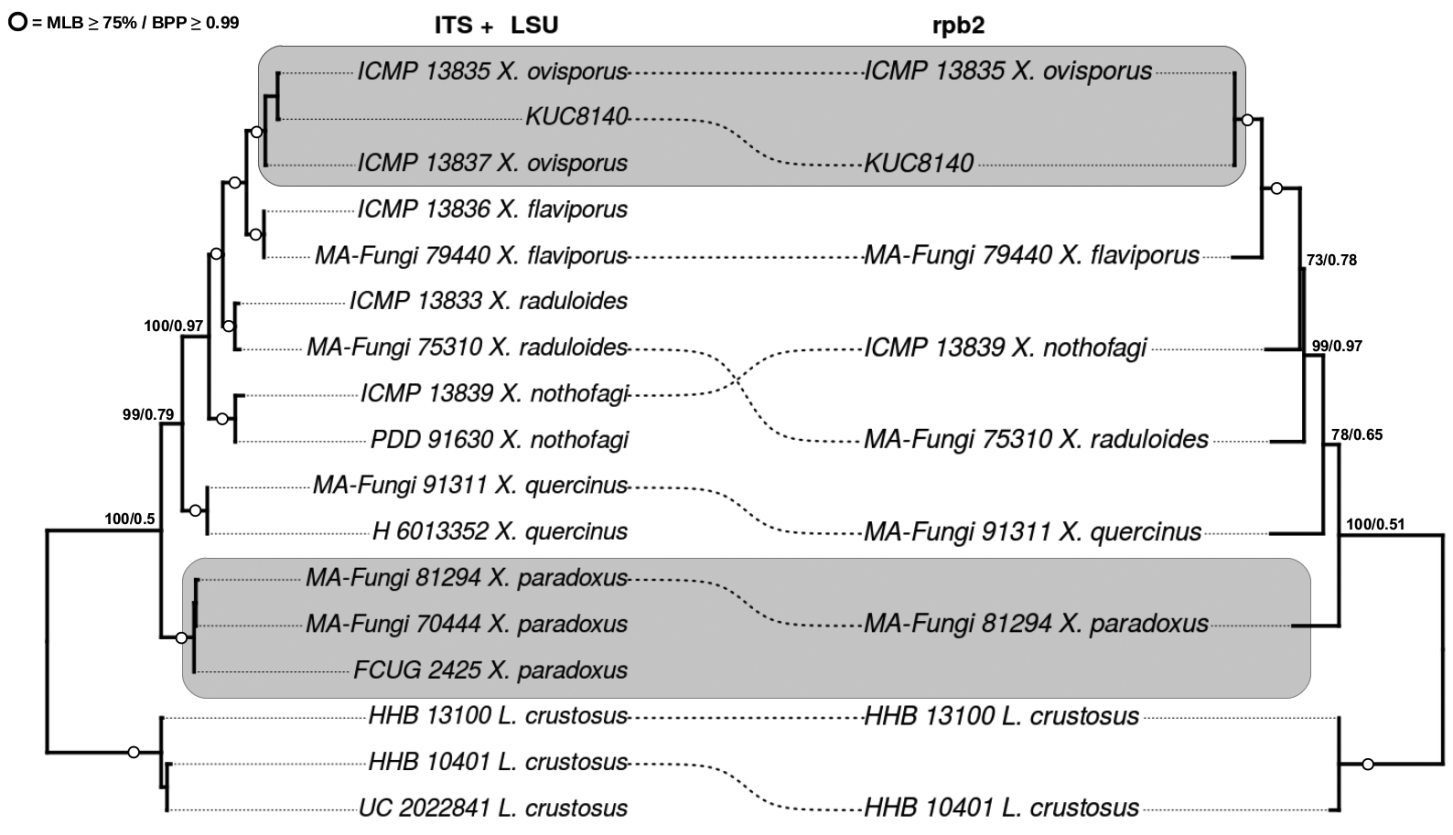
Maximum likelihood trees for ITS+LSU (left) and *rpb*2 (right) regions of *Xylodon* species. In order to assess genealogical concordance, dotted lines link the position of the same specimen in both trees. Grey boxes indicate the position of KUC8140 strain with *Xylodonovisporus* and the position of *X.paradoxus*. Numbers over branches are maximum likelihood bootstrap (MLB) values and posterior probabilities (BPP). Voucher numbers and species names are indicated in Table 1.

The ITS+LSU dataset was 1193 characters long (ITS = 594; LSU = 599) and the *rpb*2 dataset was 647 characters long. The results of phylogenetic analyses of ITS+LSU and *rpb*2 datasets are summarised in Fig. 1, using *phytools* R package (Revell 2012). Each phylogram represents the best tree produced from the RAxML analysis. All effective sample sizes from BEAST analyses were higher than 200 for all parameters. Those clades with Maximum likelihood bootstrap (MLB) percentages ≥ 75% and Bayesian posterior probabilities (BPP) ≥ 0.99 are marked with empty circles in Fig. 1. The remaining support values are represented above branches (MLB/BPP); specimen vouchers and species names are provided on the tip labels.

Our phylogenetic analyses confirmed the misidentification of S. *paradoxa* strain KUC8140, since sequences of this strain grouped in the *X.ovisporus* clade, showing a different evolutionary history from *X.paradoxus*. Therefore, *S.paradoxa* strain KUC8140, from Korea, must be identified as *Xylodonovisporus*, reported from Asia and West Pacific areas (Wu 2000, Hattori 2003). The new identity of the strain KUC8140 is also supported by geographical data, since *S.paradoxa* has a European distribution. This rectification helps to explain the biogeographical patterns of *Xylodon* and also sustains the idea that “not everything is everywhere” for wood-decay fungi (Lumbsch et al. 2008).

According to our phylogenetic analyses, *Xylodonovisporus* is the sister species of *X.flaviporus* and morphological characters confirm this relationship. The species can be discriminated by the spore size, shorter in the first one (Hattori 2003). This example accords with studies that warn about misidentifications or mislabelled vouchers in public sequence databases (Bidartondo 2008). It has been estimated that around 20% of DNA fungal sequences in the GenBank repository may have erroneous lineage assignations (Bridge et al. 2003, Nilsson et al. 2006). Assessing accuracy in GenBank and other DNA repositories is a key stage for species identification in current biodiversity analyses based on similarity of DNA sequences (Hibbett et al. 2016). It is especially important in cases like *Xylodonparadoxus*, with useful biotechnological properties since, according to Bortolus (2008), a wrong taxonomy could lead not only to inaccurate knowledge of nature, but also to important economic losses.
